# Comparative analysis of sarcopenia diagnostic criteria and their components for predicting falls in community-dwelling older adults

**DOI:** 10.1186/s12877-025-06835-3

**Published:** 2026-01-09

**Authors:** Woo Chul Son, Kyung Cheon Seo, Miji Kim, Chang Won Won, Won Kim

**Affiliations:** 1 Department of Rehabilitation Medicine, Asan Medical Center, University of Ulsan College of Medicine, 88, Olympic-Ro 43-Gil, Songpa-gu, Seoul, 05505 Republic of Korea; 2 Department of Physical Medicine and Rehabilitation, Anam Hospital, Korea University College of Medicine, Seoul, Republic of Korea; 3 Department of Health Sciences and Technology, College of Medicine, Kyung Hee University, Seoul, 02447 South Korea; 4 Department of Family Medicine, Elderly Frailty Research Center, College of Medicine, Kyung Hee University, 23, Kyungheedae-ro, Dongdaemun-gu, Seoul, Republic of Korea

**Keywords:** Sarcopenia, Gait speed, Fall, Physical performance

## Abstract

**Background:**

Sarcopenia, defined by the age-related loss of skeletal muscle mass and function, is associated with increased risk of falls, disability, and mortality in older adults. However, the strength of the relationship between sarcopenia and falls varies across studies, largely due to differences in diagnostic criteria. This study aimed to examine the associations between four widely used sarcopenia definitions and fall incidence, and to identify which components of these definitions are most predictive of falls in community-dwelling older adults.

**Methods:**

We analyzed data from 1,991 participants (aged 70–84 years; 999 men, 992 women) enrolled in the Korean Frailty and Aging Cohort Study. Sarcopenia was diagnosed using the Asian Working Group for Sarcopenia 2019 (AWGS 2019), European Working Group on Sarcopenia in Older People 2 (EWGSOP2), International Working Group on Sarcopenia (IWGS), and Foundation for the National Institutes of Health (FNIH) criteria. Appendicular lean mass was measured using dual-energy X-ray absorptiometry, muscle strength by handgrip strength (HGS), and physical performance by gait speed, five-times sit-to-stand test (5STS), and Short Physical Performance Battery (SPPB). Fall incidence was self-reported over a two-year follow-up.

**Results:**

Of the total participants, 399 (20.0%) reported at least one fall during follow-up. Fallers demonstrated significantly poorer 5STS and SPPB performance in both sexes, and lower HGS and slower gait speed in women, whereas muscle mass showed no significant association with falls in either sex. In quintile-based normalization unadjusted analyses, 5STS and SPPB remained significantly associated with falls in both sexes, and gait speed remained significant in women. Among the diagnostic criteria, slow gait speed in both sexes was significantly associated with fall risk in the unadjusted analysis.

**Conclusion:**

Sarcopenia diagnostic criteria that include physical performance measures—particularly gait speed—showed stronger associations with fall risk than those based mainly on muscle mass in unadjusted analyses. Although these associations were attenuated and became non-significant after adjustment, slow gait speed may still serve as a simple and practical indicator to screen older adults who could be at increased risk of falling. These findings suggest a potential role for physical performance assessments in sarcopenia-related fall risk evaluation.

**Supplementary Information:**

The online version contains supplementary material available at 10.1186/s12877-025-06835-3.

## Introduction

 Sarcopenia is defined as a progressive loss of skeletal muscle mass and a decline in physical function associated with aging [1, 2]. It is commonly observed in community-dwelling older adults, and numerous studies have reported its association with adverse clinical outcomes such as impaired activities of daily living, cognitive decline, increased risk of falls, and higher mortality [3, 4]. Owing to these clinical implications and the growing older adults, interest in sarcopenia has been increasing. However, there remains considerable controversy regarding which specific characteristics define sarcopenia, and different working groups have proposed various diagnostic criteria [1, 2, 5, 6]. 

Various groups, including the European Working Group and the Asian Working Group, have proposed diagnostic criteria to define sarcopenia [1, 2, 5, 6]. Sarcopenia is typically diagnosed by assessing three key domains: muscle mass, muscle strength, and physical performance. For example, the European Working Group published a guideline in 2010 (EWGSOP), defining sarcopenia as low muscle mass accompanied by low muscle strength and/or low physical performance. In 2018, a revised guideline (EWGSOP2) was introduced, redefining sarcopenia based on low muscle mass and low muscle strength alone [1]. The Asian Working Group defined sarcopenia as low muscle mass and either low muscle strength or low physical performance [2]. As above, the inconsistency among diagnostic criteria, as well as variations in measurement methods and cut-off values across the exact domains, has posed challenges to inter-study comparability and clinical implementation, such as yielding discrepant diagnostic outcomes for the same individuals and hindering meaningful comparisons in international research.

Falls are a critical clinical concern in older adults, closely linked to reduced ability to perform daily activities and increased mortality [7]. Accordingly, multiple studies have explored the relationship between sarcopenia and fall risk [3, 4]. However, as mentioned above, the diagnostic criteria for sarcopenia vary across studies, and multiple physical and physiological factors contribute to falls; therefore, the results have been inconsistent. A 2019 systematic review and meta-analysis reported that only 10 of the 22 included studies found a significantly higher fall risk among individuals diagnosed with sarcopenia [3]. To address this limitation, researchers must identify which diagnostic criteria for sarcopenia are most strongly linked to fall risk and determine which specific components best capture an individual’s likelihood of falling [8–10]. 

In this study, we analyzed baseline data from an older Asian cohort and classified participants as fallers or non-fallers based on fall events recorded two years later. Our objective was to assess whether different definitions of sarcopenia, and their respective components, determined by various cut-off values, varied between the two groups. Specifically, we aimed to identify which diagnostic domains, particularly those incorporating physical performance, most accurately reflect fall risk. We hypothesized that gait speed would show the strongest association with fall risk, given that most falls occur during transfer or gait activities and previous studies have linked gait speed to fall risk [11–13]. 

## Method

This study utilized data from the Korean Frailty and Aging Cohort Study (KFACS), a nationwide, longitudinal cohort study of older adults in South Korea. Baseline assessments took place between 2016 and 2017, followed by biennial evaluations. Participants included community-dwelling individuals aged 70 to 84, stratified by age and sex, and recruited from 10 centers spanning both urban and rural regions [14]. All research procedures adhered to the Declarations of Helsinki and Istanbul, as well as the Strengthening the Reporting of Observational Studies in Epidemiology (STROBE) guidelines.

**Fig. 1:**
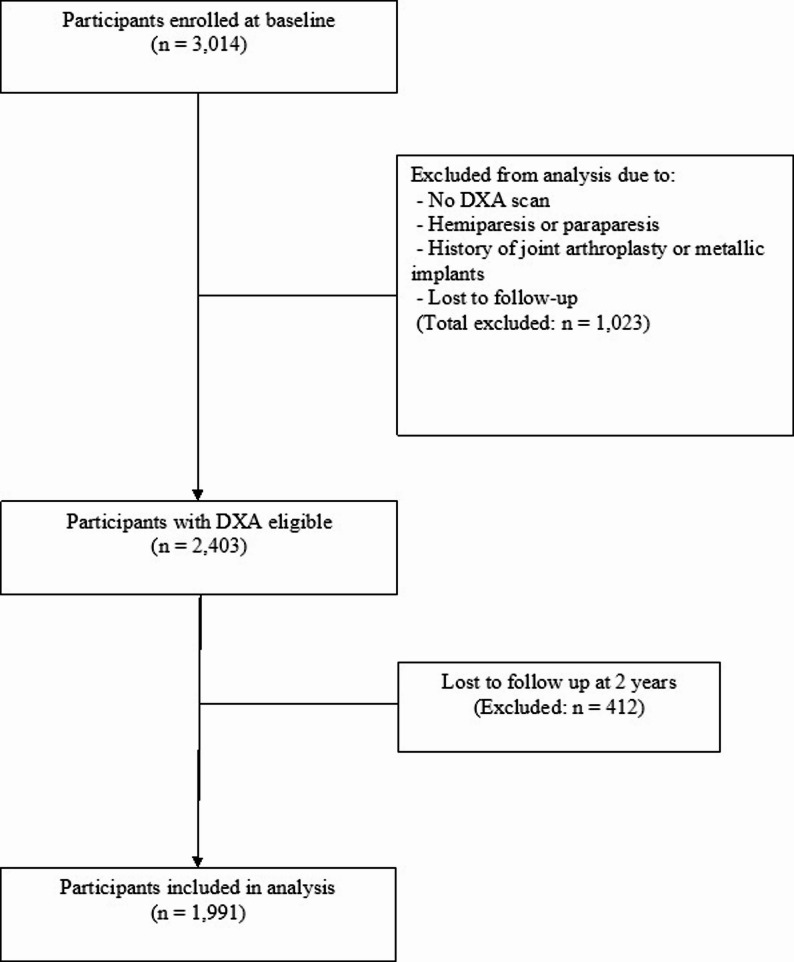
Flowchart depicting the selection process of study participants

Of the 3,014 participants initially enrolled, 2,403 who underwent dual-energy X-ray absorptiometry (DXA) were included in the analysis. Participants were excluded if they had hemiparesis or paraparesis, a history of joint arthroplasty or metallic implants in the appendicular skeleton, or were lost to follow-up (*n* = 412). Ultimately, a total of 1,991 participants (999 men and 992 women) who completed both the baseline and 2-year follow-up assessments were included in the final analysis (Fig. 1).

Demographic and clinical characteristics—including age, sex, height, body mass, body mass index (BMI), history of falls, comorbidities, and cognitive function assessed using the Korean version of the Mini-Mental State Examination(MMSE-KC) from the Consortium to Establish a Registry for Alzheimer’s Disease (CERAD) battery—were recorded at both baseline and follow-up visits. The MMSE-KC is a screening tool for global cognitive function comprising five domains: orientation (10 points), memory (6 points), attention (5 points), language ability (6 points), and comprehension and judgment (3 points). Its validity has been well established. Notably, the MMSE-KC, included in the CERAD-K Assessment Packet, was specifically modified to account for the high illiteracy rate among older Korean adults [15, 16]. This distinguishes it from the K-MMSE (or K-MMSE-2), which directly corresponds to the MMSE (or MMSE-2) and has been employed in other studies [15, 16]. 

The study protocol was approved by the Institutional Review Board of Kyung Hee University Medical Center (IRB No. 2015-12−103), and written informed consent was obtained from all participants.

### Assessment of sarcopenia components and diagnosis criteria

**Fig. 2:**
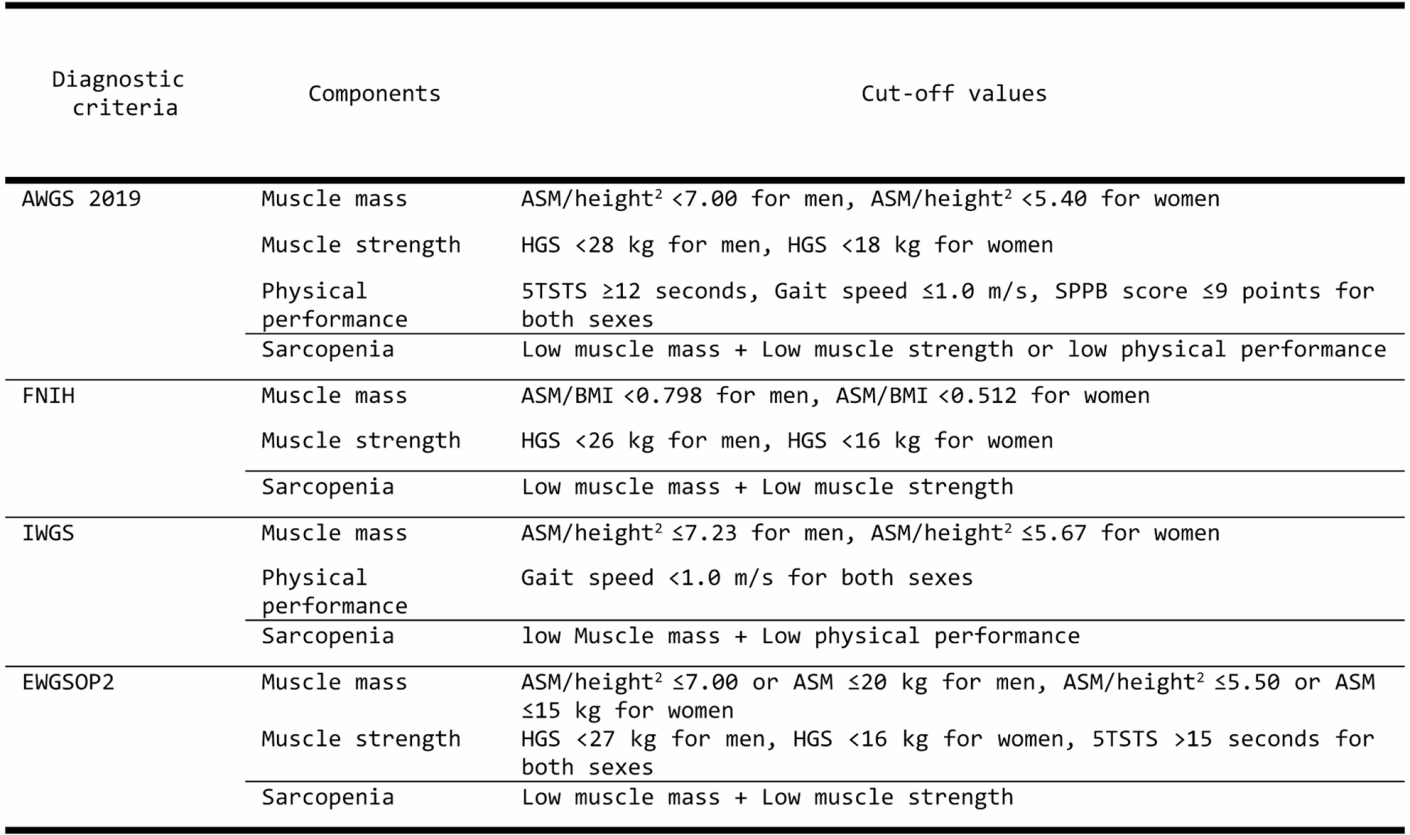
Definitions of sarcopenia and cut-off values across diagnostic criteria. ASM, Appendicular skeletal muscle mass; HGS, Handgrip strength; BMI, Body mass index; GS, Gait speed; 5TSTS, 5 times sit-to-stand test; SPPB, Short Physical Performance Battery

Sarcopenia was diagnosed using four established criteria, each incorporating distinct components and cut-off thresholds: the Asian Working Group for Sarcopenia (AWGS) 2019, EWGSOP2, the International Working Group on Sarcopenia (IWGS), and the Foundation for the National Institutes of Health (FNIH). We selected these four definitions (AWGS 2019, EWGSOP2, IWGS, and FNIH) because they include guidelines developed by working groups from Europe, Asia, and North America, as well as an international consensus framework, thereby representing the most globally recognized and widely applied diagnostic criteria. These frameworks assess three core domains: low muscle mass, weak muscle strength, and low physical performance. While all four criteria required low muscle mass as a foundational element, they varied in their inclusion of muscle strength and physical performance measures, as well as in the specific cut-off values applied (Fig. 2). For example, AWGS 2019 defines sarcopenia as low muscle mass in conjunction with either weak muscle strength or low physical performance, whereas EWGSOP2 identifies it as low muscle mass combined with weak muscle strength.

Muscle mass was quantified via DXA. Appendicular skeletal muscle mass (ASM) was calculated as the total lean soft tissue mass in the arms and legs. To meet the respective criteria, ASM was normalized by height squared (ASM/ht²), body mass (ASM/wt), or BMI (ASM/BMI), depending on the diagnostic framework used.

Muscle strength was measured using handgrip strength (HGS), assessed with a digital handgrip dynamometer (JAMAR, Bolingbrook, IL, USA) [17]. Each participant performed two trials with both hands, and the highest value obtained was used for analysis.

Physical performance was evaluated using three functional tests: gait speed, the five-times sit-to-stand test (5TSTS), and the Short Physical Performance Battery (SPPB) [18]. Gait speed was measured over a 4-meter walkway, and the average speed was calculated. The coefficient of variation was 9.2%, and the intraclass correlation coefficient indicated good reliability at 0.86 (95% CI, 0.84–0.87). In the 5TSTS, the time required to rise from a seated position to a complete stand five times, without the use of arms, was recorded. As only a single trial was performed, the absence of repeated measurements represents a limitation of this study. The SPPB comprises three components—standing balance, gait speed, and chair stand test—each scored from 0 to 4 points, yielding a total score ranging from 0 to 12.

### Falls

Fall incidence was determined through self-reported responses to the question: “Have you had a fall in the past year?” In the follow-up survey, individuals who reported one or more falls in the previous 12 months were classified as fallers, whereas those who reported no falls were classified as non-fallers.

### Statistical analysis

Continuous variables were expressed as means ± standard deviations, and categorical variables as frequencies and percentages. The homogeneity of variances was tested using Levene’s test.

Participants were first stratified by sex, and between-group comparisons were conducted to examine sex-related differences in the prevalence of sarcopenia and its diagnostic components according to different diagnostic criteria. Subsequently, within each sex, subgroup analyses were conducted to compare fallers and non-fallers in terms of baseline characteristics and physical performance measures. Continuous variables were analyzed using independent t-tests or Mann–Whitney U tests, as appropriate, and categorical variables using chi-square or Fisher’s exact tests. The prevalence of sarcopenia and its components was further compared according to each diagnostic criterion.

Variables that showed significant differences between fallers and non-fallers were then entered into logistic regression models to assess their association with fall incidence. To standardize the comparison of continuous performance measures, participants were categorized into quintiles. The lowest quintile, representing the poorest performance, was compared against the upper four quintiles combined. Univariate analyses were first performed, followed by multivariable models adjusted for age, BMI, comorbidities, cognitive function (MMSE-KC), and fall history. Comorbidities were categorized as ≤ 1 or ≥ 2, MMSE-KC scores were dichotomized using a cutoff value of 23, with scores ≤ 23 indicating mild cognitive impairment and scores ≥ 24 indicating normal cognition, based on widely accepted criteria [19]. Fall history was treated as a binary variable.

To further evaluate the diagnostic performance of existing criteria, receiver operating characteristic (ROC) curves were generated, and the area under the curve (AUC) was calculated. Cut-off values were determined using Youden’s index and compared with those defined in existing diagnostic criteria. Finally, logistic regression analyses were used to assess the association between falls and each component based on the derived cut-off values. All statistical analyses were conducted using SPSS version 23.0 (SPSS Inc., Chicago, IL, USA).

## Results

Table 1 summarizes the prevalence of sarcopenia and its diagnostic components according to various operational definitions, showing that sarcopenia defined by AWGS 2019 and FNIH was significantly more common in men than in women. However, the prevalence of low physical performance, as determined by IWGS and AWGS 2019, was significantly higher in women. Specifically, slow gait speed (defined as < 1.0 m/s), indicating low physical performance according to the IWGS criteria, was observed in 331 women (33%) compared to 228 men (23%).

**Table 1:** Prevalence of sarcopenia according to different diagnostic criteria and their components

Diagnostic criteria	Components	Men (*n* = 999)	Women (*n* = 992)	Total (*n* = 1991)	*P*
AWGS 2019	Low muscle mass	482 (48)	280 (28)	762 (38)	0.000*
	Low muscle strength	207 (21)	189 (19)	396 (20)	0.369
	Low physical performance	390 (39)	533 (54)	923 (46)	0.000*
	Sarcopenia	263 (26)	189 (19)	452 (23)	0.000*
FNIH	Low muscle mass	496 (50)	291 (29)	787 (40)	0.000*
	Low muscle strength	114 (11)	100 (10)	214 (11)	0.348
	Sarcopenia	77 (8)	46 (5)	123 (6)	0.005*
IWGS	Low muscle mass	587 (59)	433 (44)	1020 (51)	0.000*
	Low physical performance	228 (23)	331 (33)	559 (28)	0.000*
	Sarcopenia	152 (15)	159 (16)	311 (16)	0.622
EWGSOP2	Low muscle mass	657 (66)	797 (80)	1,454 (73)	0.000*
	Low muscle strength	216 (22)	235 (24)	451 (23)	0.284
	Sarcopenia	164 (16)	194 (20)	358 (18)	0.071

Values are presented as a number (%)

* *p*-value <0.05, the chi-square test or Fisher’s exact test

Table 2 summarizes the baseline characteristics of participants stratified by faller and non-faller status, indicating that 399 of 1,991 individuals (153 men and 246 women) experienced at least one fall during the past year. Regarding the components of sarcopenia, there were no significant differences in ASM or its normalized indices between fallers and non-fallers, despite muscle mass being a fundamental element in the definition of sarcopenia. However, 5TSTS and SPPB scores were significantly worse in fallers of both sexes, and among women, HGS and gait speed were also significantly lower in fallers.

**Table 2:** Baseline characteristics and sarcopenia components of the study population

Characteristics	Men (*n* = 999)				Women (*n* = 992)			
	Non-faller (*n* = 846)	Faller (*n* = 153)	*p*	Cohen’s d	Non-faller (*n* = 746)	Faller (*n* = 246)	*p*	Cohen’s d
Demographics								
Age (years)	76.21 ± 3.91	76.90 ± 3.65	0.045*	−0.182	75.27 ± 3.88	75.83 ± 3.92	0.054	−0.144
Height (cm)	164.85 ± 5.47	164.65 ± 5.94	0.684	0.035	152.30 ± 5.19	151.30 ± 5.24	0.009*	0.192
Body mass (kg)	65.24 ± 8.89	64.55 ± 8.10	0.374	0.081	56.72 ± 7.56	56.36 ± 7.74	0.519	0.047
BMI (kg/m^2^)	23.99 ± 2.90	23.80 ± 2.58	0.446	0.069	24.42 ± 2.75	24.59 ± 2.88	0.412	−0.060
History of previous falls (%)	103 (12.17)	48 (31.37)	0.000*		142 (19.03)	81 (32.93)	0.000*	
MMSE	26.51 ± 2.80	25.94 ± 2.91	0.021*	0.200	25.61 ± 3.25	24.92 ± 3.44	0.005*	0.206
Sarcopenia Component								
ASM (kg)	19.22 ± 2.61	18.91 ± 2.80	0.176	0.115	13.46 ± 1.86	13.40 ± 1.81	0.651	0.033
ASM/Height^2^ (kg/m^2^)	7.07 ± 0.84	6.96 ± 0.88	0.162	0.128	5.80 ± 0.71	5.85 ± 0.71	0.317	−0.070
ASM/body mass	0.30 ± 0.03	0.29 ± 0.03	0.338	0.333	0.24 ± 0.03	0.24 ± 0.03	0.826	0
ASM/BMI	0.81 ± 0.10	0.80 ± 0.11	0.370	0.095	0.55 ± 0.07	0.55 ± 0.08	0.350	0
Handgrip strength (kg)	32.63 ± 5.71	31.74 ± 5.27	0.073	0.162	21.40 ± 3.88	20.57 ± 4.14	0.005*	0.206
5-times sit-to-stand-test (s)	10.39 ± 3.17	11.01 ± 2.95	0.026*	−0.202	11.63 ± 3.88	12.42 ± 4.63	0.021*	−0.185
Gait speed (m/s)	1.19 ± 0.26	1.14 ± 0.29	0.060	0.182	1.11 ± 0.23	1.05 ± 0.24	0.000*	0.255
SPPB score	11.23 ± 1.15	10.95 ± 1.33	0.007*	0.225	10.79 ± 1.48	10.44 ± 1.72	0.002*	0.218

Values are presented as a number (%) or the mean ± standard deviation

*BMI* body mass index, *MMSE *Mini-Mental State Examination, *ASM *Appendicular skeletal muscle mass, *SPPB *Short Physical Performance Battery

*p-value <0.05, the independent t-test or Mann–Whitney U test for continuous variables, and the chi-square test for categorical variables

Table 3 summarizes the associations between handgrip strength, physical performance measures, and fall risk, showing that both 5TSTS and SPPB scores in both sexes and HGS and gait speed in women were significantly related to falls in the unadjusted analysis. After adjustment for confounders, gait speed remained significantly associated with fall risk in women. When comparing the lowest quintile, gait speed in women and both the 5TSTS and SPPB in both sexes were significantly associated with fall risk in unadjusted models, but these associations disappeared after adjustment.

**Table 3:** Logistic regression analysis for the association between low handgrip strength, physical performance, and falls

	Men (*n*=999)			Women (*n*=992)		
	Odds	95% CI	*p*	Odds	95% CI	*p*
Unadjusted						
HGS	1.029	0.997–1.059	0.073	1.050	1.012–1.089	0.009*
5TSTS	1.059	1.006–1.114.006.114	0.029*	1.076	1.023–1.131	0.004*
SPPB	1.192	1.047–1.339	0.008*	1.135	1.036–1.242	0.006*
Gait speed	1.916	0.973–3.788	0.060	3.448	1.809–6.579	0.000*
HGS (lowest quintile)	1.170	0.771–1.776	0.460	1.357	0.968–1.901	0.077
5TSTS (lowest quintile)	1.541	1.033–2.299	0.034*	1.544	1.098–2.172	0.013*
SPPB score (lowest quintile)	1.598	1.087–2.349	0.017*	1.509	1.065–2.139	0.021*
Gait speed (lowest quintile)	1.406	0.938–2.108	0.099	1.592	1.132–2.237	0.007*
Adjusted						
HGS	1.004	0.970–1.038.970.038	0.835	1.042	1.000–1.083.000.083	0.042*
5TSTS	1.030	0.975–1.089.975.089	0.287	1.035	0.999–1.073.999.073	0.057
SPPB	1.081	0.935–1.250.935.250	0.291	1.093	0.989–1.206.989.206	0.082
Gait speed	2.445	0.665–2.724.665.724	0.409	2.660	1.285–5.495.285.495	0.008*
HGS (lowest quintile)	0.909	0.576–1.434.576.434	0.682	1.143	0.792–1.651.792.651	0.475
5TSTS (lowest quintile)	1.245	0.808–1.918.808.918	0.321	1.253	0.869–1.807.869.807	0.227
Gait speed (lowest quintile)	1.150	0.743–1.781.743.781	0.530	1.330	0.912–1.939.912.939	0.138
SPPB score (lowest quintile)	1.228	0.804–1.876.804.876	0.341	1.311	0.903–1.902.903.902	0.155

Unadjusted and all-adjusted logistic regression analyses evaluating the associations between handgrip strength and physical performance (assessed as continuous values and lowest quintile groups) and fall incidence in men and women. The adjusted models were controlled for age, BMI, MMSE score, previous fall history, and comorbidities. Odds ratios (ORs) and 95% confidence intervals (CIs) are shown

*OR* odds ratio, *CI *confidence interval, *HGS *Handgrip strength, *5TSTS *5 times sit-to-stand test, *SPPB *Short Physical Performance Battery

*p-value <0.05, logistic regression analysis

Table 4 summarizes the prevalence of sarcopenia and its components among fallers and non-fallers across different diagnostic criteria, showing that the AWGS 2019 and IWGS definitions identified a significantly higher prevalence of sarcopenia in fallers than in non-fallers among men. However, no significant difference in sarcopenia prevalence was found in women under any definition. Notably, the prevalence of slow gait speed, as included in the AWGS 2019 and IWGS definitions, was significantly higher in fallers than non-fallers in both sexes. In contrast, low muscle mass was not significantly different between fallers and non-fallers under any diagnostic criteria in either sex.

**Table 4:** Prevalence of sarcopenia and its components according to different diagnostic criteria

		Men (*n*=999)			Women (*n*=992)		
		Non-faller (*n*=846)	Faller (*n*=153)	*p*	Non-faller (*n*=746)	Faller (*n*=246)	*P*
AWGS 2019	Low muscle mass	403 (48)	79 (52)	0.380	215 (29)	65 (26)	0.514
	Weak handgrip strength	171 (20)	36 (24)	0.386	136 (18)	53 (22)	0.262
	Low physical performance	310 (37)	80 (52)	0.000*	386 (52)	147 (60)	0.032*
	Gait speed <1.0 m/s	179 (21)	49 (32)	0.004*	232 (31)	99 (40)	0.010*
	5TSTS ≥12 seconds	220 (26)	51 (33)	0.060	285 (38)	105 (43)	0.229
	SPPB score ≤9	72 (9)	19 (12)	0.127	129 (17)	59 (24)	0.024*
	Sarcopenia	210 (25)	53 (35)	0.013*	137 (18)	52 (21)	0.350
FNIH	Low muscle mass	413 (49)	83 (54)	0.220	210 (28)	81 (33)	0.170
	Weak handgrip strength	96 (11)	18 (12)	0.890	68 (9)	32 (13)	0.087
	Sarcopenia	64 (8)	13 (8)	0.741	29 (4)	17 (7)	0.056
IWGS	Low muscle mass	491 (58)	96 (63)	0.286	324 (43)	109 (44)	0.824
	Gait speed<1.0m/s	179 (21)	49 (32)	0.004*	232 (31)	99 (40)	0.010*
	Sarcopenia	116 (14)	36 (24)	0.002*	112 (15)	47 (19)	0.134
EWGSOP2	Low muscle mass	554 (65)	103 (67)	0.711	594 (80)	203 (83)	0.355
	ASM	535 (63)	100 (65)	0.649	594 (80)	203 (83)	0.355
	ASM/ht2	403 (48)	79 (52)	0.380	257 (34)	80 (33)	0.588
	Low muscle strength	179 (21)	37 (24)	0.455	165 (22)	70 (28)	0.047*
	Weak handgrip strength	131 (15)	30 (20)	0.231	68 (9)	32 (13)	0.087
	5TSTS >15 seconds	64 (8)	12 (8)	1.000	119 (16)	51 (21)	0.097
	Sarcopenia	138 (16)	26 (17)	0.906	136 (18)	58 (24)	0.078

Category	Diagnostic criteria	Men (*n*=999)			Women (*n*=992)		
		Non-faller (*n*=846)	Faller (*n*=153)	*p*	Non-faller (*n*=746)	Faller (*n*=246)	*P*
Muscle mass	AWGS 2019	403 (48)	79 (52)	0.380	215 (29)	65 (26)	0.514
	FNIH	413 (49)	83 (54)	0.220	210 (28)	81 (33)	0.170
	IWGS	491 (58)	96 (63)	0.286	324 (43)	109 (44)	0.824
	EWGSOP2	554 (65)	103 (67)	0.711	594 (80)	203 (83)	0.355
Muscle strength	AWGS 2019	171 (20)	36 (24)	0.386	136 (18)	53 (22)	0.262
	FNIH	96 (11)	18 (12)	0.890	68 (9)	32 (13)	0.087
	EWGSOP2	179 (21)	37 (24)	0.455	165 (22)	70 (28)	0.047*
Physical performance	Gait speed <1.0 m/s	179 (21)	49 (32)	0.004*	232 (31)	99 (40)	0.010*
	5TSTS >15 seconds	64 (8)	12 (8)	1.000	119 (16)	51 (21)	0.097
	5TSTS ≥12 seconds	220 (26)	51 (33)	0.060	285 (38)	105 (43)	0.229
	SPPB score ≤9	72 (9)	19 (12)	0.127	129 (17)	59 (24)	0.024*
Sarcopenia	AWGS 2019	210 (25)	53 (35)	0.013*	137 (18)	52 (21)	0.350
	FNIH	64 (8)	13 (8)	0.741	29 (4)	17 (7)	0.056
	IWGS	116 (14)	36 (24)	0.002*	112 (15)	47 (19)	0.134
	EWGSOP2	138 (16)	26 (17)	0.906	136 (18)	58 (24)	0.078

Comparison of sarcopenia prevalence and its components based on AWGS 2019, FNIH, IWGS, and EWGSOP2 criteria in fallers and non-fallers by sex. Weak handgrip strength was defined according to the criteria presented in Fig. 2

Values are presented as a number (%)

*ASM* Appendicular skeletal muscle mass, *Ht *Height, *5TSTS *5 times sit-to-stand test, *SPPB *Short Physical Performance Battery

*p-value <0.05, the chi-square test or Fisher’s exact test

Table 5 further evaluates the associations between falls and gait speed, 5TSTS, SPPB, and sarcopenia, based on the observed prevalence differences in Table 4. In unadjusted models, slow gait speed, IWGS-defined sarcopenia and AWGS 2019-defined sarcopenia were significantly associated with falls in men. In women, slow gait speed and low SPPB score (≤ 9) were significantly associated with falls. After adjusting for confounders, no significant associations with fall risk were observed.

**Table 5:** Logistic regression analysis of the association between sarcopenia, low physical performance defined by diagnostic criteria, and falls

	Men (*n*=999)			Women (*n*=992)		
	Odds	95% CI	*p*	Odds	95% CI	*p*
Unadjusted						
Physical performance						
5TSTS ≥12 seconds	1.430	0.988-2.070	0.058	1.205	0.899–1.614	0.213
5TSTS >15 seconds	1.138	0.611-2.122	0.683	1.378	0.956–1.986	0.085
Gait speed <1.0 m/s	1.756	1.204–2.561	0.003*	1.492	1.107–2.011	0.009*
SPPB score ≤9	1.524	0.890–2.610	0.124	1.509	1.065–2.139	0.021*
Sarcopenia						
AWGS 2019	1.605	1.111–2.319	0.012*	1.192	0.833–1.704	0.337
FNIH	1.135	0.609–2.115	0.691	1.835	0.990–3.401	0.054
IWGS	1.936	1.270–2.952	0.002*	1.337	0.918–1.947	0.130
EWGSOP2	1.050	0.663–1.663	0.834	1.384	0.977–1.960	0.067
Adjusted						
Physical performance						
5TSTS ≥12 seconds	1.211	0.811-1.808	0.348	1.108	0.815-1.506	0.512
5TSTS >15 seconds	0.928	0.483-1.784	0.823	1.241	0.848-1.817	0.266
Gait speed <1.0 m/s	1.352	0.895-2.043	0.152	1.295	0.936-1.792	0.118
SPPB score ≤9	0.947	0.527-1.702	0.857	1.311	0.903-1.902	0.155
Sarcopenia						
AWGS 2019	1.373	0.902-2.091	0.139	1.131	0.770-1.661	0.530
FNIH	0.905	0.466-1.756	0.767	1.520	0.796-2.903	0.205
IWGS	1.478	0.934-2.340	0.095	1.223	0.822-1.819	0.322
EWGSOP2	0.792	0.480-1.308	0.362	1.251	0.869-1.802	0.228

Unadjusted and adjusted logistic regression analyses evaluating the associations between physical performance measures and sarcopenia (as defined by AWGS 2019, FNIH, IWGS, and EWGSOP2 criteria) and fall incidence in men and women. The adjusted models were controlled for age, BMI, MMSE score, previous fall history, and comorbidities. Odds ratios (ORs) and 95% confidence intervals (CIs) are shown

*OR* odds ratio, *CI *confidence interval, *5TSTS *5 times sit-to-stand test, *SPPB *Short Physical Performance Battery

*p-value <0.05, logistic regression analysis

Finally, the Supplementary Table and Figure present the ROC curve analysis used to determine the cut-off values. Although the AUC values were below 0.6 and thus lack strong clinical implication, the cut-off values for gait speed were 1.00 m/s in men and 1.09 m/s in women. Notably, even after adjusting for all confounders, gait speed below the cut-off value remained significantly associated with fall risk, particularly in women.

## Discussion

This study aimed to identify the most suitable diagnostic criteria for sarcopenia in evaluating fall risk among older Asian adults. We also examined which components of sarcopenia were most strongly linked to falls and determined cut-off values at which fall risk significantly increased. While prior research has established the connection between sarcopenia, physical performance, and fall risk, few studies have directly compared several diagnostic criteria and individual components to assess their predictive validity [7]. Consistent with our hypothesis, the comparative analysis suggested that gait speed tended to show the association with fall risk among the diagnostic components examined, particularly when analyzed as a continuous variable, although it did not remain significant when categorized using diagnostic cut-offs. Interpretation should be cautious given the potential recall bias from self-reported fall data collected two years after baseline.

As previously established, fall risk is associated with factors such as fall history, cognitive function, muscle strength, and physical performance [7, 20]. Similar trends were observed in our findings, as shown in Table 2. Both the 5TSTS and the SPPB revealed significant performance differences between fallers and non-fallers among men and women, with non-fallers consistently outperforming fallers. Gait speed and HGS were significantly associated with fall status in women. In men, these two indicators showed only borderline significance, although a similar trend toward lower function in fallers was observed. This sex difference may be partly explained by the larger number of fallers in women (approximately 100 more than men) and the overall higher functional status of men in the cohort. In contrast, muscle mass did not differ significantly between fallers and non-fallers in either sex, which supports the view that muscle mass alone may not adequately reflect muscular function or physical performance [21, 22]. It also aligns with current trends in sarcopenia research and clinical practice, which prioritize muscle strength and performance over mass in diagnostic and risk stratification frameworks [1, 2]. 

In Table 3, all unadjusted physical performance indicators, analyzed both as continuous variables and by quintiles, showed significant associations with falls among women, with similar trends observed in men. After adjustment, only gait speed as the continuous variable remained significantly associated with fall risk, particularly in women, whereas other physical performance measures lost their significance. These findings suggest that the most predictive tests extend beyond simple measures of muscle mass or strength. Instead, they capture lower extremity function as well as abilities critical to fall risk in daily life, including neuromuscular control, balance, and coordination [11, 23, 24]. These results support previous evidence indicating that gait speed or SPPB reflects overall physiological function and correlates closely with both survival and fall risk [11, 23]. In addition, among the quintile-based standardized comparisons, gait speed yielded the highest odds ratio for fall risk in women. The 5TSTS primarily reflects lower extremity strength and may not adequately capture other fall-related domains [24, 25]. The SPPB, a categorical scoring system, may exhibit a ceiling effect in relatively healthy older adults, reducing its ability to detect subtle declines in function [23]. In contrast, gait speed, measured as a continuous variable, offers greater sensitivity across multiple physical domains, thus making it a more comprehensive measure of functional impairment associated with fall risk. However, since the 5TSTS was performed only once in this study, the possibility of measurement error cannot be completely excluded. These findings have practical implications for clinicians and exercise professionals, suggesting that physical performance measures such as gait speed can be used to guide individualized exercise prescription and fall prevention strategies in sarcopenic and frail older adults, consistent with the mobility or functional oriented training approach [26, 27]. In particular, the quintile-based comparison revealed that individuals in the poorest performance group may require more intensive and prolonged rehabilitation interventions, along with closer monitoring to prevent further functional decline or falls.

When comparing the prevalence of sarcopenia between fallers and non-fallers according to various diagnostic criteria, only slow gait speed demonstrated a statistically significant difference in both men and women (Table 4). This trend was further supported by unadjusted logistic regression analyses, in which slow gait speed consistently showed significant odds ratios for fall risk (Table 5). However, after adjustment for confounders, none of the predefined cut-off values remained significant. Given that multiple unadjusted comparisons were conducted, some of the observed associations could have arisen by chance, and the absence of formal correction for multiple testing should be considered when interpreting these findings. Although several physical performance tests were associated with falls (Tables 2 and 3), these results suggest that physical performance measures, including gait speed, may be more appropriately used as continuous or supportive indicators rather than relying on a single cut-off value to predict fall risk. Although the AUC values were low, limiting their clinical applicability, ROC curve analyses were additionally performed as a post-hoc approach to explore potential cut-off values. When comparing the Youden’s index–derived cut-off values from ROC curve analyses with those defined by existing diagnostic criteria (e.g., AWGS 2019, IWGS), the thresholds for most tests except for gait speed were generally stricter in our study (Supplementary Figs. 1 and 2; Supplementary Table 1). For instance, previous studies have proposed 5TSTS cut-off values for fall risk as 9.9 s for men and 10.53 s for women, and the cut-off value for gait speed has been reported as 0.8 m/s, which approximately corresponds to an SPPB score of 9. Comparisons indicate that fall risk-based thresholds generally demand higher levels of physical function than those used in sarcopenia diagnoses, such as 12 s for the 5TSTS or ≤ 9 points for SPPB [28, 29]. This discrepancy likely stems from the intent behind sarcopenia definitions, which prioritize sensitivity for identifying individuals with pathological conditions, often at the expense of specificity. Correspondingly, Table 4 shows that the prevalence of low SPPB scores (≤ 9) and prolonged 5TSTS (≥ 15 s) among fallers was lower than that of slow gait speed, reinforcing this explanation. Nevertheless, the ROC-based cut-off analyses should be regarded as exploratory, given their limited discriminatory power.

Among various sarcopenia definitions, AWGS 2019 and IWGS, which both include gait speed as a diagnostic criterion, revealed significant differences in sarcopenia prevalence between fallers and non-fallers, particularly in men. Notably, although the study population comprised Asian adults, IWGS demonstrated greater statistical significance than AWGS 2019, possibly due to differences in how physical performance is weighted with each framework. Accordingly, our results may contribute to future revisions or refinements of sarcopenia diagnostic guidelines by highlighting the importance of selecting appropriate physical performance tools that more accurately reflect overall functional status.

A key strength of the present study is its comprehensive comparison of multiple sarcopenia diagnostic frameworks, enabling the identification of the most effective tool for fall risk assessment in affected individuals. Previous studies often relied on a single definition of sarcopenia or physical performance within specific populations, without evaluating the relative effectiveness of various criteria. Additionally, many prior investigations included heterogeneous groups, such as nursing home residents or hospitalized patients, rather than focusing on community-dwelling older adults [3, 7]. 

By analyzing a well-defined and relatively large cohort of community-dwelling older adults, our study partially compensated for some of these limitations. It provided a direct comparison of the most widely used diagnostic criteria within this population. This approach strengthens the generalizability and clinical relevance of our findings for both healthcare providers and community-based interventions.

This study has several limitations. First, although we included several confounding factors, not all potential covariates related to fall risk were accounted for in the analysis [30]. However, because the aim was to evaluate fall risk primarily based on physical performance tests—which are known to capture overall functional status even in the absence of detailed clinical information—we conducted analyses mainly based on univariate models. Second, the follow-up survey conducted two years after baseline collected data on falls occurring within the preceding 12 months. Despite efforts to limit recall bias, this design inherently introduces temporal incongruity and a high risk of misclassification, as fall events were recalled long after the baseline assessments. Such a delay may have caused substantial underreporting and incomplete capture of fall episodes during the follow-up period. Furthermore, all fall information was self-reported, without details on fall frequency or severity, which further undermines the reliability and clinical interpretability of the findings. Third, although we evaluated numerous performance measures in relation to fall risk, we did not apply a formal multiple testing correction. The possibility of type I error inflation cannot be completely excluded and should be considered when interpreting the results. Finally, this study included only community-dwelling older adults in Asia. Therefore, caution is warranted when generalizing the findings to populations of different ethnic backgrounds or residential settings, such as institutionalized older adults.

## Conclusion

In this study, physical performance components—particularly gait speed—showed the strongest associations with fall risk among various sarcopenia diagnostic criteria. Although these associations were attenuated after adjustment for major confounders, gait speed may serve as a practical and supportive indicator for fall risk screening within sarcopenia assessment frameworks.

## Supplementary Information

Supplementary Material 1.

## Data Availability

The data analyzed in this study are available from the Korean Frailty and Aging Cohort Study (KFACS) upon reasonable request and with permission from the study’s steering committee. Due to ethical restrictions and institutional guidelines, the raw data are not publicly available.
