# Supplementary material for: Comparative analysis of sarcopenia diagnostic criteria and their components for predicting falls in community-dwelling older adults
Source: BMC Geriatr. 2026 Jan 9;26:333. doi: 10.1186/s12877-025-06835-3 (PMC12983790; doi:10.1186/s12877-025-06835-3)
Supplement: Supplementary file 1 — Supplementary Material 1. [file 12877_2025_6835_MOESM1_ESM.docx]

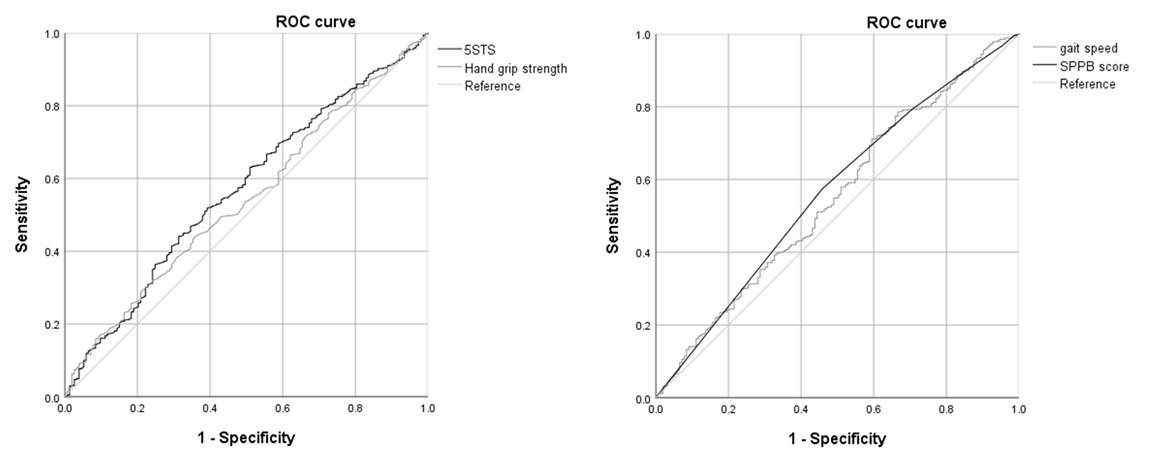


**Cut off value**
Gait speed: 1.00
SPPB: 11.5

**Cut off value**
5TSTS: 10.23
HGS: 33.5

**Supplementary Figure 1** ROC curve for the association between hand grip strength, physical performance and falls in males.
Cut-off values were determined using Youden’s index.

ROC, Receiver operating characteristic; 5TSTS, 5 times sit-to-stand test; SPPB, Short Physical Performance Battery; HGS, Handgrip strength


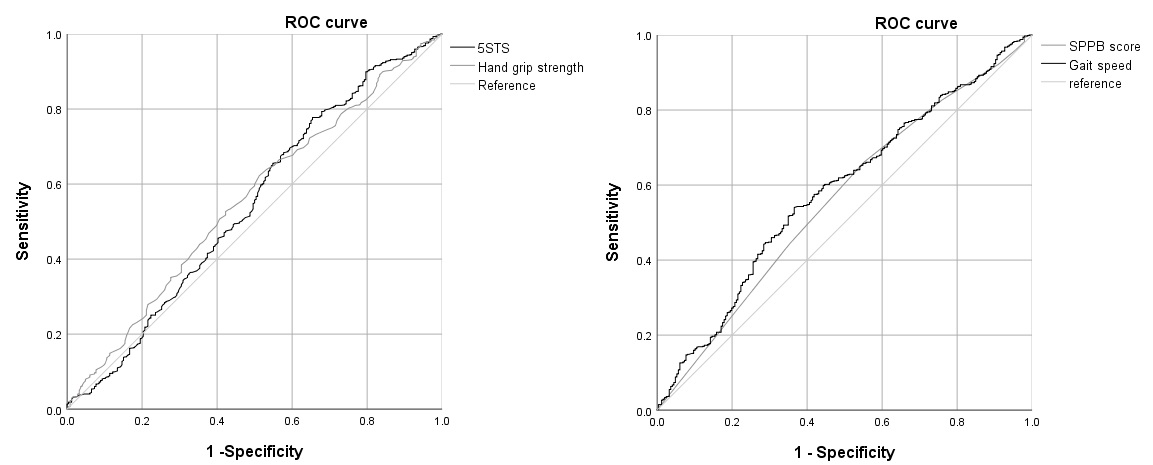


**Cut off value**
Gait speed: 1.09
SPPB: 10.5

**Cut off value**
5TSTS: 11.53
HGS: 20.3

**Supplementary Figure 2** ROC curve for the association between hand grip strength, physical performance and falls in females.
Cut-off values were determined using Youden’s index.

ROC, Receiver operating characteristic; 5TSTS, 5 times sit-to-stand test; SPPB, Short Physical Performance Battery; HGS, Handgrip strength

**Supplementary Table 1** ROC curve of Handgrip Strength and Physical Performance for Falls

|  |  | Men (n=999) | | | |  | Women (n=992) | | | |
| --- | --- | --- | --- | --- | --- | --- | --- | --- | --- | --- |
|  |  | Cut-off value | AUC | 95% CI | *p* |  | Cut-off value | AUC | CI | *p* |
| HGS (Kg) |  | 33.5 | 0.542 | 0.494–0.590 | 0.099 |  | 20.3 | 0.556 | 0.514–0.597 | 0.009* |
| 5TSTS(s) |  | 10.23 | 0.568 | 0.518–0.618 | 0.007* |  | 11.53 | 0.544 | 0.500–0.587 | 0.040* |
| Gait speed(s) |  | 1.00 | 0.550 | 0.500–0.601 | 0.047* |  | 1.09 | 0.583 | 0.519–0.602 | 0.004* |
| SPPB score |  | 11.5 | 0.565 | 0.515–0.615 | 0.010* |  | 10.5 | 0.583 | 0.542–0.624 | 0.000* |

*p-value <0.05, logistic regression analysis. The adjusted models were controlled for age, BMI, MMSE score, previous fall history, and comorbidities.

The cut-off value was obtained by Youden’s index.

AUC, Area under curve; HGS, Handgrip strength; 5TSTS, 5 times sit-to-stand test; SPPB, Short Physical Performance Battery.

**Supplementary Table 2** Logistic Regression Analysis of the Association Between Low Handgrip Strength and Physical Performance Defined by ROC Curve Cut-off and Falls

|  |  | Men (n=999) | | |  | Women (n=992) | | |
| --- | --- | --- | --- | --- | --- | --- | --- | --- |
|  |  | Odds | 95% CI | *p* |  | Odds | 95% CI | *p* |
| Unadjusted (Male, female) |  |  |  |  |  |  |  |  |
| HGS (Kg) (<33.5, <20.3) |  | 1.405 | 0.983–2.008 | 0.062 |  | 1.567 | 1.172–2.095 | 0.002* |
| 5TSTS(s) (>10.23, >11.53) |  | 1.635 | 1.156–2.314 | 0.005* |  | 1.816 | 1.327–2.486 | 0.000* |
| Gait speed (m/s) (<1.00, <1.09) |  | 1.756 | 1.204–2.561 | 0.003* |  | 2.037 | 1.514–2.740 | 0.000* |
| SPPB score (<11.5, <10.5) |  | 1.593 | 1.127–2.252 | 0.008* |  | 1.586 | 1.182–2.127 | 0.002* |
| Adjusted (Male, female) |  |  |  |  |  |  |  |  |
| HGS (Kg) (<33.5, <20.3) |  | 1.143 | 0.774-1.688 | 0.502 |  | 1.439 | 1.061-1.952 | 0.019* |
| 5TSTS(s) (>10.23, >11.53) |  | 1.529 | 1.036-2.255 | 0.032* |  | 1.212 | 0.885-1.660 | 0.232 |
| Gait speed (m/s) (<1.00, <1.09) |  | 1.352 | 0.895-2.043 | 0.152 |  | 1.882 | 1.373-2.581 | <0.001* |
| SPPB score (<11.5, <10.5) |  | 1.374 | 0.947-1.996 | 0.095 |  | 1.473 | 1.076-2.016 | 0.016* |

Unadjusted and adjusted logistic regression analyses evaluating the associations between physical performance measures (as defined by ROC curve) and fall incidence in men and women. The adjusted models were controlled for age, BMI, MMSE score, previous fall history, and comorbidities. Odds ratios (ORs) and 95% confidence intervals (CIs) are shown.

*p-value <0.05, logistic regression analysis.

HGS, Handgrip strength; 5TSTS, 5 times sit-to-stand test; SPPB, Short Physical Performance Battery; ROC, Receiver operating characteristic.
